# Exposure to Static Magnetic Field Stimulates Quorum Sensing Circuit in Luminescent *Vibrio* Strains of the Harveyi Clade

**DOI:** 10.1371/journal.pone.0100825

**Published:** 2014-06-24

**Authors:** Adelfia Talà, Domenico Delle Side, Giovanni Buccolieri, Salvatore Maurizio Tredici, Luciano Velardi, Fabio Paladini, Mario De Stefano, Vincenzo Nassisi, Pietro Alifano

**Affiliations:** 1 Dipartimento di Scienze e Tecnologie Biologiche ed Ambientali, Università del Salento, Lecce, Italy; 2 Dipartimento di Matematica e Fisica “Ennio De Giorgi”, Università del Salento INFN – Lecce, Lecce, Italy; 3 Dipartimento di Scienze Ambientali, Seconda Università di Napoli, Caserta, Italy; State Key Laboratory of Pathogen and Biosecurity, Beijing Institute of Microbiology and Epidemiology, China

## Abstract

In this study, the evidence of electron-dense magnetic inclusions with polyhedral shape in the cytoplasm of Harveyi clade *Vibrio* strain PS1, a bioluminescent bacterium living in symbiosis with marine organisms, led us to investigate the behavior of this bacterium under exposure to static magnetic fields ranging between 20 and 2000 Gauss. When compared to sham-exposed, the light emission of magnetic field-exposed bacteria growing on solid medium at 18°C ±0.1°C was increased up to two-fold as a function of dose and growth phase. Stimulation of bioluminescence by magnetic field was more pronounced during the post-exponential growth and stationary phase, and was lost when bacteria were grown in the presence of the iron chelator deferoxamine, which caused disassembly of the magnetic inclusions suggesting their involvement in magnetic response. As in luminescent *Vibrio* spp. bioluminescence is regulated by quorum sensing, possible effects of magnetic field exposure on quorum sensing were investigated. Measurement of mRNA levels by reverse transcriptase real time-PCR demonstrated that *luxR* regulatory gene and *luxCDABE* operon coding for luciferase and fatty acid reductase complex were significantly up-regulated in magnetic field-exposed bacteria. In contrast, genes coding for a type III secretion system, whose expression was negatively affected by LuxR, were down-regulated. Up-regulation of *luxR* paralleled with down-regulation of small RNAs that mediate destabilization of *luxR* mRNA in quorum sensing signaling pathways. The results of experiments with the well-studied *Vibrio campbellii* strain BB120 (originally classified as *Vibrio harveyi*) and derivative mutants unable to synthesize autoinducers suggest that the effects of magnetic fields on quorum sensing may be mediated by AI-2, the interspecies quorum sensing signal molecule.

## Introduction

All living organisms are continuously exposed to weak magnetic fields at the Earth's surface. However, in modern society humans have the opportunity to experience moderate or relatively strong static magnetic fields (SMF), for example through the use of diagnostic magnetic resonance imaging equipment in the medical field, and certain types of industrial processes. This raised a concern about the possible risk on human health of exposure to moderate strength SMF. While now it seems clear that exposure to SMF can induce biological changes, the precise effects provoked and the underlying mechanisms are not well known. Three physical interactions of SMF with biological systems have been postulated: electro-dynamic induction with ionic conduction currents, magneto-mechanical interaction and electro-spin interaction [1]–[3]. In this latter, SMF exposure can affect the conversion between singlet and triplet states of radicals and change the radical pair recombination rate [2].

Over the last fifty years, the influence of SMF on biological systems has been investigated in a variety of model organisms. In general, studies investigating bacteria as opposed to eukaryotic cell cultures, tissues, and animals are less frequent [3]–[4]. In bacteria, moderate strength SMF were shown to affect growth and survival [5]–[10] but the results remained inconclusive. It is usually argued that the controversial or inconsistent results obtained so far are mainly due to the different experimental protocols that have been used.

In this study we have investigated the effect of SMF on bioluminescence and quorum sensing in Harveyi clade *Vibrio* strains. Bacterial luminescence has emerged as an extremely useful and versatile reporter technology to monitor stressful conditions. It provides a sensitive, non-destructive, and real-time assay that allows for temporal and spatial measurement [11]. Moreover, the biochemical pathway leading to bioluminescence and the control mechanisms have been carefully characterized in several bacteria [12]–[13]. All these features make luminescent bacteria an excellent model to study the influence of SMF on biological systems. By using a Harveyi clade *Vibrio* strain isolated from a marine hydrozoon [14]–[15] harboring magnetic crystals in their cytoplasm, we have found that bioluminescence is affected by exposure to moderate strength SMF. When exposed to a SMF an enhancement of the luminous intensity emitted by the cultures growing at 18°C on solid medium was observed during the stationary phase as a function of the magnetic field strength. This effect was suppressed by treatment with the iron chelator deferoxamine suggesting involvement of iron in magnetic sensing.

In *Vibrio* spp. bioluminescence is regulated by quorum sensing (QS) [12], the communication circuit that many bacteria use to sense population density and regulate, in a coordinate fashion, a diverse array of physiological activities that are presumably productive only when groups of cells act in concert [13]. QS regulation of Harveyi clade *Vibrio spp.* is rather complex. These bacteria produce and respond to three autoinducers (AIs): HAI-1 (*N*-[β-hydroxybutyryl] homoserine lactone), a species-specific AI [24]–[25], CAI-1 ([*S*]-3-hydroxytridecan-4-one), a genus-specific signal [16]–[17], and AI-2 ([2*S*,4*S*]-2-methyl-2,3,3,4-tetrahydroxytetrahydrofuran borate), an interspecies signal [18]–[20].

HAI-1, CAI-1, and AI-2 are detected by the membrane-bound, two-component sensors LuxN, CqsS, and LuxQ, respectively [21]–[24]. At low cell density, when the AIs concentrations are low, the sensors act as kinases and transfer phosphate to the histidine phospho-transfer protein LuxU, which in turn, passes the phosphate to the response regulator LuxO [22]–[26]. Phospho-LuxO, together with sigma 54, activates the expression of the genes encoding five regulatory small RNAs (sRNAs) called Quorum Regulatory RNA 1–5 (Qrr1–5) [27]–[28]. In conjunction with the RNA chaperone Hfq, the sRNAs destabilize the mRNA encoding the master quorum-sensing regulator LuxR [12]. Because LuxR is required for transcription of the *luxCDABE* operon, coding for the luciferase and the fatty acid reductase complex, under low-cell-density conditions luminous bacteria produce reduced levels of luminescence that may be below detection. At high cell density, the presence of the AIs converts the sensors from kinases to phosphatases, resulting in dephosphorylation and inactivation of LuxO, no Qrr expression, and stabilization of the *luxR* mRNA, leading to LuxR production. Thus, under high-cell-density conditions, light is produced.

Our results demonstrate that *luxR* and the *luxCDABE* operon were significantly up-regulated in response to SMF, and they are consistent with a model by which exposure to static SMF may up-regulate LuxR expression by inhibiting small RNAs-mediated destabilization of *luxR* mRNA.

## Results

### Harveyi clade *Vibrio* strain PS1 contains unusual electron-dense magnetic inclusions

**Figure 1:**
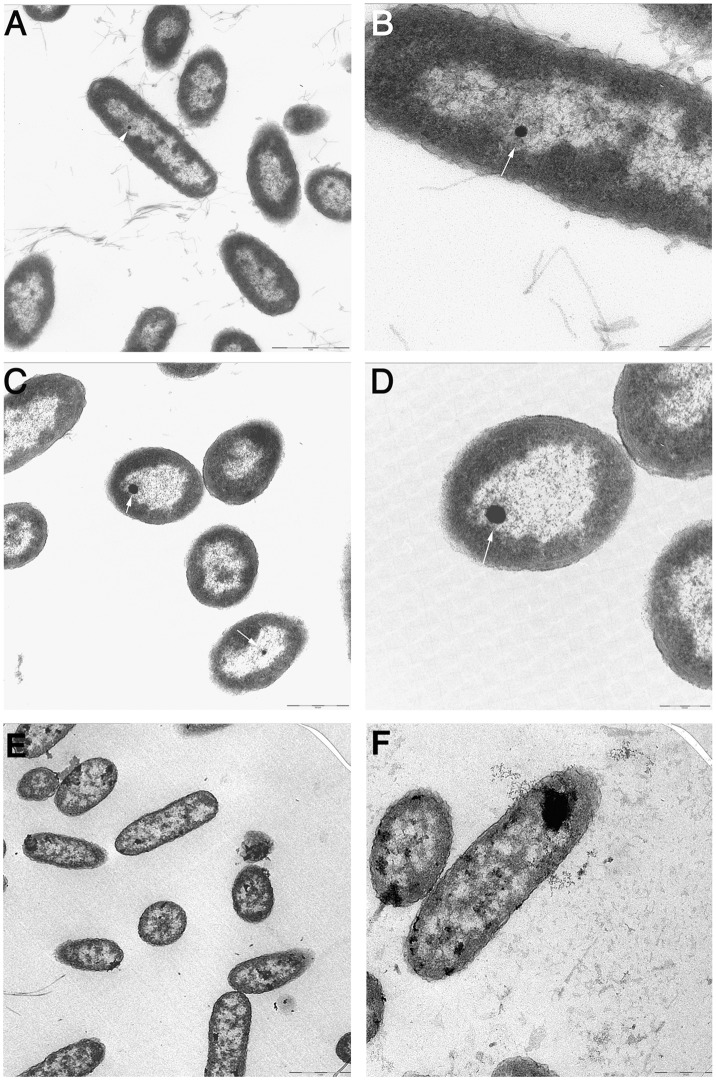
Transmission electron microscopy (TEM) analysis of *Vibrio* sp. PS1. In panels A–D, note the presence, in the nucleoid area, of the electron-dense inclusions about 10–50 nm in size exhibiting a polyhedral shape (arrows). Panels B and D are enlargement of regions of panels A and C, respectively. In panels E and F bacteria were grown in the presence of 31.2 µM deferoxamine that caused disassembly of the electron-dense inclusions with polyhedral shape and the appearance of leading to accumulation of electron-dense amorphous aggregates. Bars represent 1 µm in A and E, 0.5 µm in C and F, and 0.2 µm in B and D.

**Figure 2:**
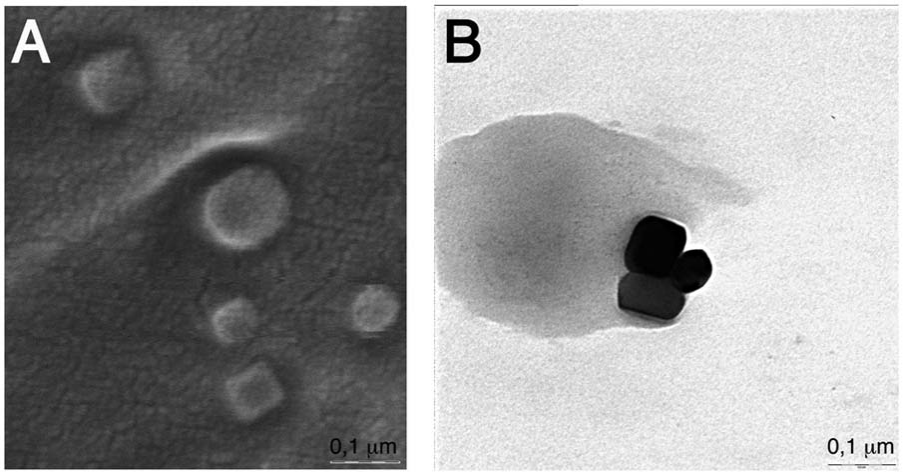
Images of purified magnetic inclusions. Purified magnetic inclusions were analyzed by scanning electron microscopy (SEM) (A) or transmission electron microscopy (TEM) (B). Note the tendency of the inclusions to stick together confirming their magnetic nature. Bars represent 0.1 µm in A and B.

PS1 is a luminescent *Vibrio* strain belonging to the Harveyi clade, which was recently isolated from a marine hydrozoon [14]–[15]. In the course of a study dealing with the ultra-structure of this bacterial isolate, transmission electron microscopy (TEM) images showed intracellular electron dense inclusions about 10–50 nm in size (Figure 1A–D). These inclusions exhibited a polyhedral shape and were typically located in the nucleoid area. They did not appear to be surrounded by membrane or membrane-like structure and were never arranged in a chain. The inclusions (or at least some of them) are magnetic matter as they could be purified by magnetic separation and observed by TEM and scanning electron microscopy (SEM) (Figure 2A–B).

The electron density of the inclusions led to hypothesize the presence of iron. However, we failed to define their chemical nature due scarcity of the material that could be recovered. Thus we attempted to gain more information by growing the bacteria in the presence of the iron chelator deferoxamine. As expected, treatment with deferoxamine caused growth delay and reduction of luminous intensity as a function of dose (Figure S1). Interestingly, TEM analysis demonstrated that treatment with the iron chelator inhibited formation of the inclusions leading to accumulation of electron-dense amorphous aggregates suggesting the presence of iron in the electron-dense inclusions (Figure 1E–F).

### Bioluminescence is stimulated by SMF exposure

**Figure 3:**
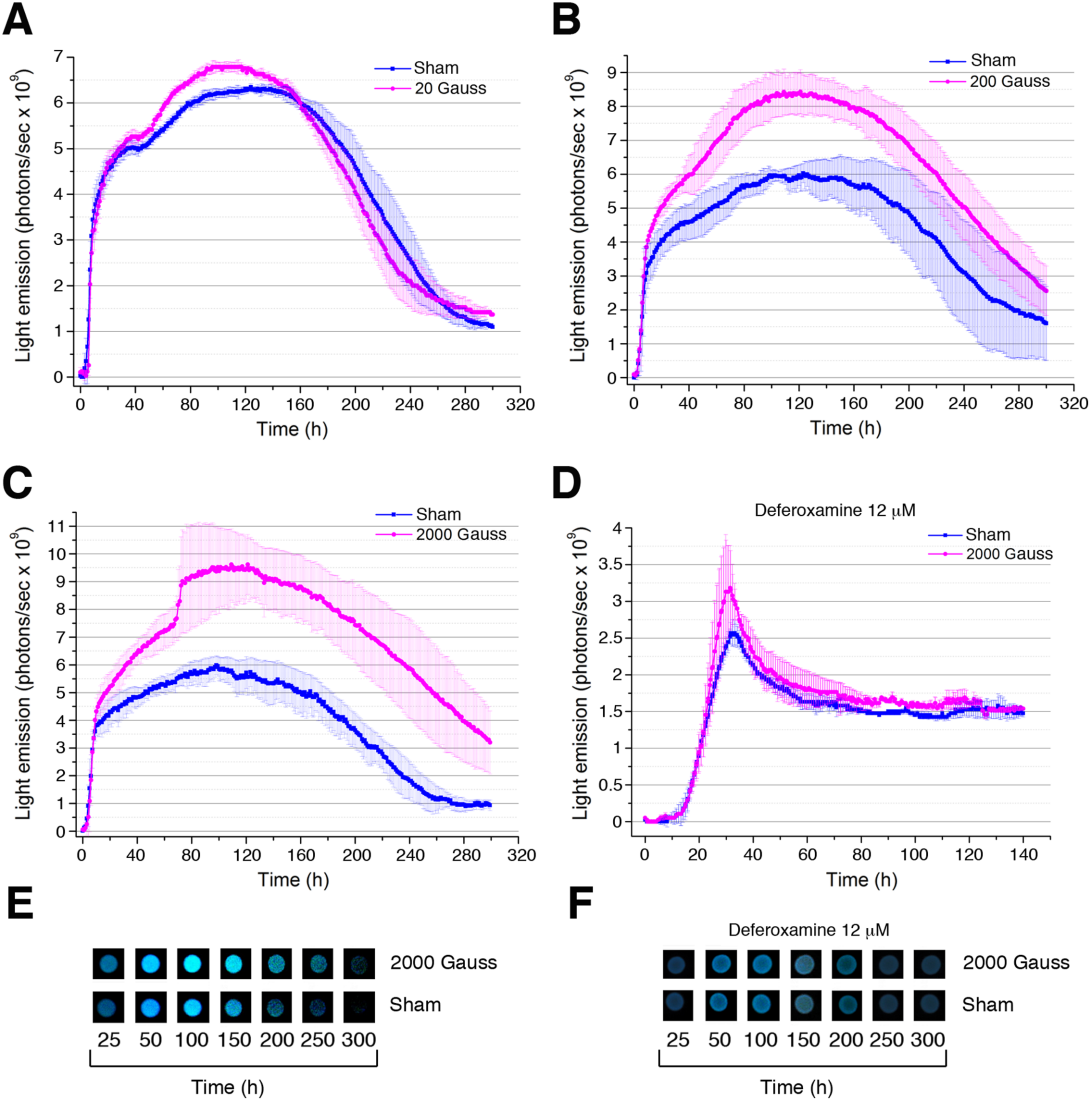
Light emission monitoring during growth of *Vibrio* sp. PS1. In panels A–C, light emission of SMF-exposed (20, 200 or 2000 Gauss) or sham-exposed *Vibrio* sp. PS1 was monitored during growth on nutrient agar 1.5% containing 3% NaCl. In panel D bacteria were cultivated in the presence of 12 µM deferoxamine and SMF-exposed (2000 Gauss) or sham-exposed. In each panel data represent means values and standard deviations (bars) of at least three measurements of different cultures for each treatment. In panel E–F, light emission of SMF-exposed (2000 Gauss) or sham-exposed *Vibrio* sp. PS1 was monitored by photographs either in the absence (E) or the presence of 12 µM deferoxamine (F).

The evidence of magnetic crystals in the cytoplasm of *Vibrio* sp. PS1 led us to investigate the behavior of this bacterium under exposure to SMF. To monitor bioluminescence, bacteria were spotted onto agar plates and exposed to SMF of 20, 200 and 2000 Gauss or sham-exposed during their growth in a climate chamber under nearly constant temperature (18.0±0.1°C), in the dark. Luminescence was measured over a period of 350 h by using the apparatus showed in Figure S2–S4. *Vibrio* sp. PS1 exhibited an intense luminescence showing a peak at 470 nm and a shoulder near 500 nm (Figure S5). The luminescence of the sham-exposed samples increased rapidly during the first 12 h and more slowly during the next 24 h reaching a plateau at around 36 h. After 54 h up to 96 h a detectable increase of luminous intensity was also recorded (Figure 3A). When compared to sham-exposed bacteria, the light emission of SMF-exposed bacteria was not significantly affected at 20 Gauss, but it increased after 12 h as a function of the magnetic field intensity at 200 and 2000 Gauss with a more pronounced enhancement after 54 h of cultivation (Figure 3B–C). The enhancement of the light emission in bacteria exposed to the SMF of 2000 Gauss was also visible in photographs (Figure 3E). In contrast, the enhancement was not observed when the bacteria were grown in the presence of the iron chelator deferoxamine (12 µM) suggesting an involvement of the magnetic crystals and/or iron metabolism (Figure 3D and 3F).

**Figure 4:**
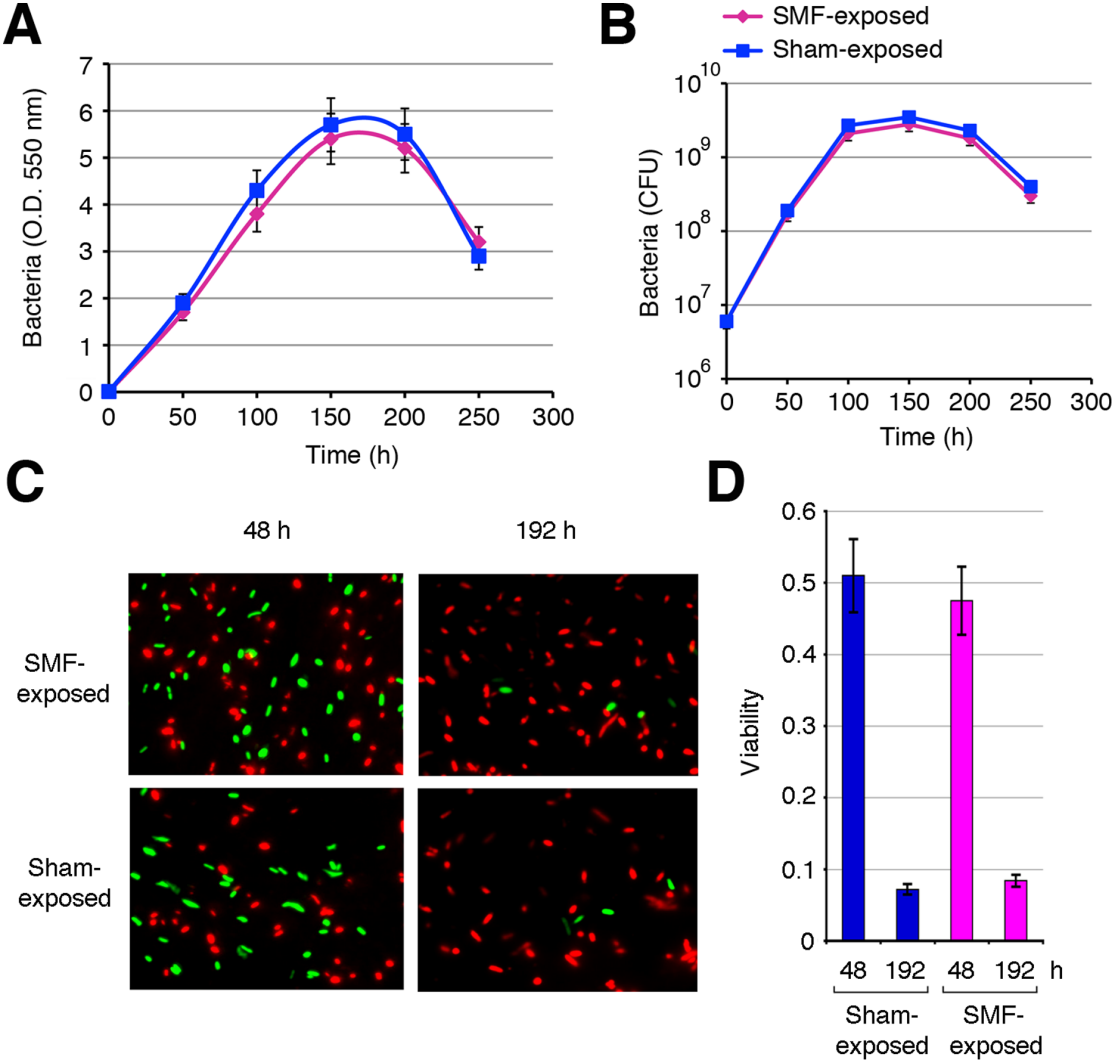
Growth and viability of SMF-exposed and sham-exposed *Vibrio* sp. PS1. Ten µl of a 1 O.D. (550 nm) suspension of *Vibrio* sp. PS1 were spotted at the centre of 3% NaCl nutrient agar plates and incubated at 18°C either in the absence (sham-exposed) or in the presence of SMF (2000 Gauss) (SMF-exposed). Bacteria were recovered at different time intervals and re-suspended in 1 ml of 3% NaCl nutrient broth. Growth and viability were evaluated by determining the O.D. (550 nm) (A), the number of CFU/ml (B) and by using a dead/live staining as detailed in the Materials and Methods section. Data are shown as mean ± standard deviation from at least three independent experiments.

The stimulation of bioluminescence was not due to an effect of SMF on bacterial growth or viability. Indeed, assessment of optical density (O.D.) of recovered bacteria (Figure 4A) and viability by using either the CFU method (Figure 4B) or a dead/live staining (Figure 4C–D) demonstrated the absence of any significant differences between SMF-exposed (2000 Gauss) and sham-exposed bacteria. The stimulation was not even due to a direct effect of SMF on the photochemical reaction catalyzed by the bacterial luciferase because the luminous intensity of the bacteria grown for 48 h without SMF (20, 200 or 2000 Gauss) did not change in a short time (as expected if a direct effect of SMF on the photochemical reaction were involved) following exposure to SMF (data not shown). We thus believed that gene regulation could be involved.

### Regulation of QS-controlled *lux* genes is influenced by SMF exposure

**Figure 5:**
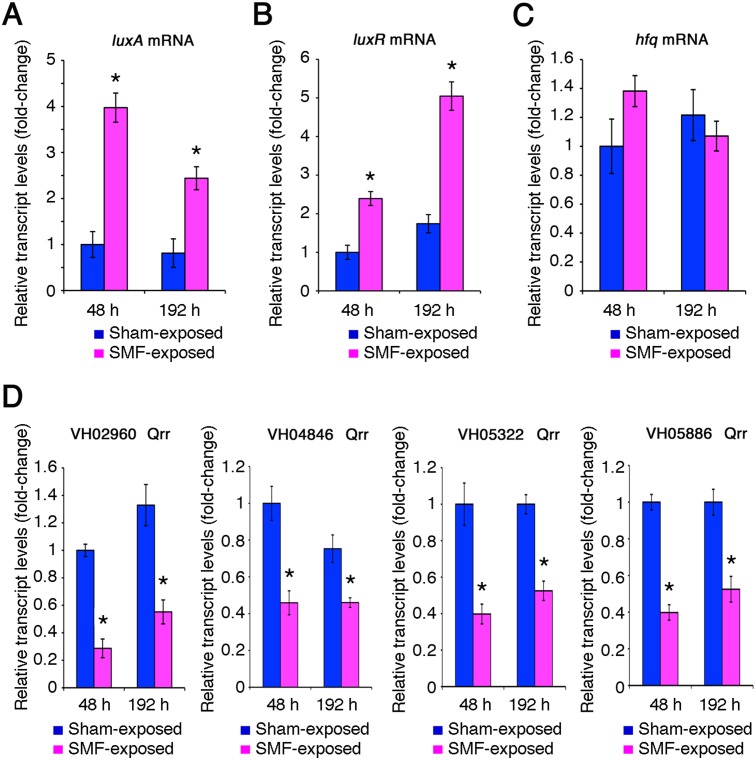
Transcript levels of *luxA* and regulatory genes. Transcript levels of *luxA* (A), *luxR* (B), *hfq* (C), and VH02969, VH04846, VH05322 and VH05886 genes coding for Quorum Regulatory RNA (Qrr) (D) were measured in SMF-exposed or sham-exposed *Vibrio* sp. PS1 after 48 or 192 h of growth on nutrient agar 1.5% containing 3% NaCl. Results were normalized to 16S rRNA levels. Transcript levels of sham-exposed bacteria grown for 48 h are assumed equal to 1. Each real-time RT-PCR experiment was repeated three times using RNA samples extracted from replicated bacterial cultures for each treatment, and means and standard deviations (bars) were determined. Asterisks indicate statistically significant differences (p<0.01) between values of SMF-exposed and sham-exposed bacteria at the same time intervals.

Expression of *luxA* gene of the *luxCDABE* operon was monitored in SMF-exposed (2000 Gauss) and sham-exposed bacteria by real time RT-PCR. Results demonstrated a significant increase in *luxA* mRNA levels in SMF-exposed bacteria both at 48 and 192 h (Figure 5A and Table S1). Such an increase was parallel with an increase in mRNA levels of the master regulatory gene *luxR* (Figure 5B and Table S1). To gain insight into the mechanism underlying this behavior, levels of *hfq* mRNA and possible homologues of four of the five Qrrs were measured. Indeed, there is evidence that *luxR* mRNA levels are negatively regulated at a post-transcriptional level by a mechanism involving Qrrs in conjunction with the RNA chaperone Hfq [12]. The Qrrs encoding genes examined in this study were VIBHAR_02960 (VH02960), VIBHAR_04846 (VH04846), VIBHAR_05322 (VH05322) and VIBHAR_05886 (VH05886), derived from Harveyi clade *Vibrio* strain BB120 (also known as ATCC BAA-1116).

Comparison of *hfq* mRNA levels between SMF-exposed and sham-exposed bacteria did not yield any significant difference at corresponding time points (Figure 5C and Table S1). In contrast, the results of real time RT-PCR demonstrated that Qrrs levels were significantly reduced in bacteria exposed to SMF (Figure 5D and Table S1). By assuming a general conservation in quorum signaling pathways between strain PS1 and the reference strain BB120, our results suggest that exposure to SMF may up-regulate LuxR expression by reducing Qrrs levels.

### Regulation of other QS-controlled genes is also influenced by SMF exposure

The *luxCDABE* operon is not the exclusive target of QS regulation in Harveyi clade *Vibrio* strains. LuxR, directly or indirectly, controls the expression of several virulence factors including a type III secretion system (TTS) [29], extracellular toxin [30], metalloprotease [31] and a siderophore [27]. TTS systems are specialized secretion machineries used by many gram-negative plant and animal pathogens to inject virulence factors/effectors directly into the cytoplasm of eukaryotic host cells with which they are associated [32]–[33].

**Figure 6:**
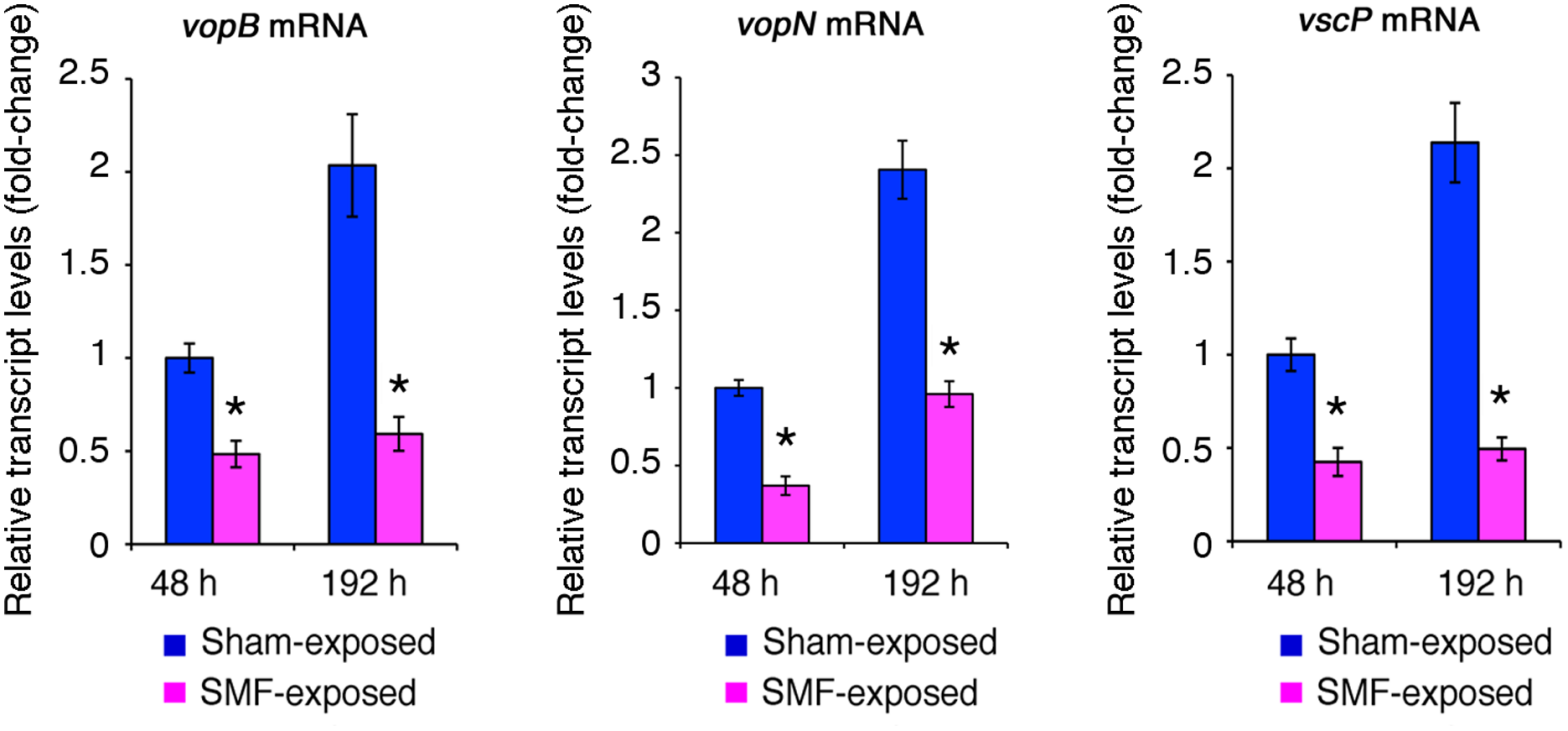
Transcript levels of type III secretion system (TTS)-encoding genes. Transcript levels of TTS-encoding genes *vopB*, *vopN* and *vscP* were measured in SMF-exposed or sham-exposed *Vibrio* sp. PS1 after 48 or 192 h of growth on nutrient agar 1.5% containing 3% NaCl. Results were normalized to 16S rRNA levels. Transcript levels of sham-exposed bacteria grown for 48 h are assumed equal to 1. Each real-time RT-PCR experiment was repeated three times using RNA samples extracted from replicated bacterial cultures for each treatment, and means and standard deviations (bars) were determined. Asterisks indicate statistically significant differences (p<0.01) between values of SMF-exposed and sham-exposed bacteria at the same time intervals.

We thus decided to analyze also the expression of other genes of the LuxR regulon in SMF-exposed and sham-exposed bacteria. In strain BB120 expression of three TTS-encoding gene clusters, *vcrG-hyp-vcrH-vopBD*, *vopN-vcr1-vcr2-vscXY-vcrDR* and *vscNOPQRSTU*, requires an intact QS signal transduction cascade, and is repressed at high cell density by secreted AIs [29]. *vopB*, *vopN* and *vscP* mRNA levels were thus monitored in SMF-exposed and sham-exposed bacteria. The results of real time RT-PCR demonstrated that *vopB*, *vopN* and *vscP* mRNA levels were significantly reduced in bacteria exposed to SMF (Figure 6), suggesting that global regulation of the QS circuit was responsive to SMF.

### Bioluminescence is not stimulated by SMF in the AI-2-defective Δ*luxS* mutant

**Figure 7:**
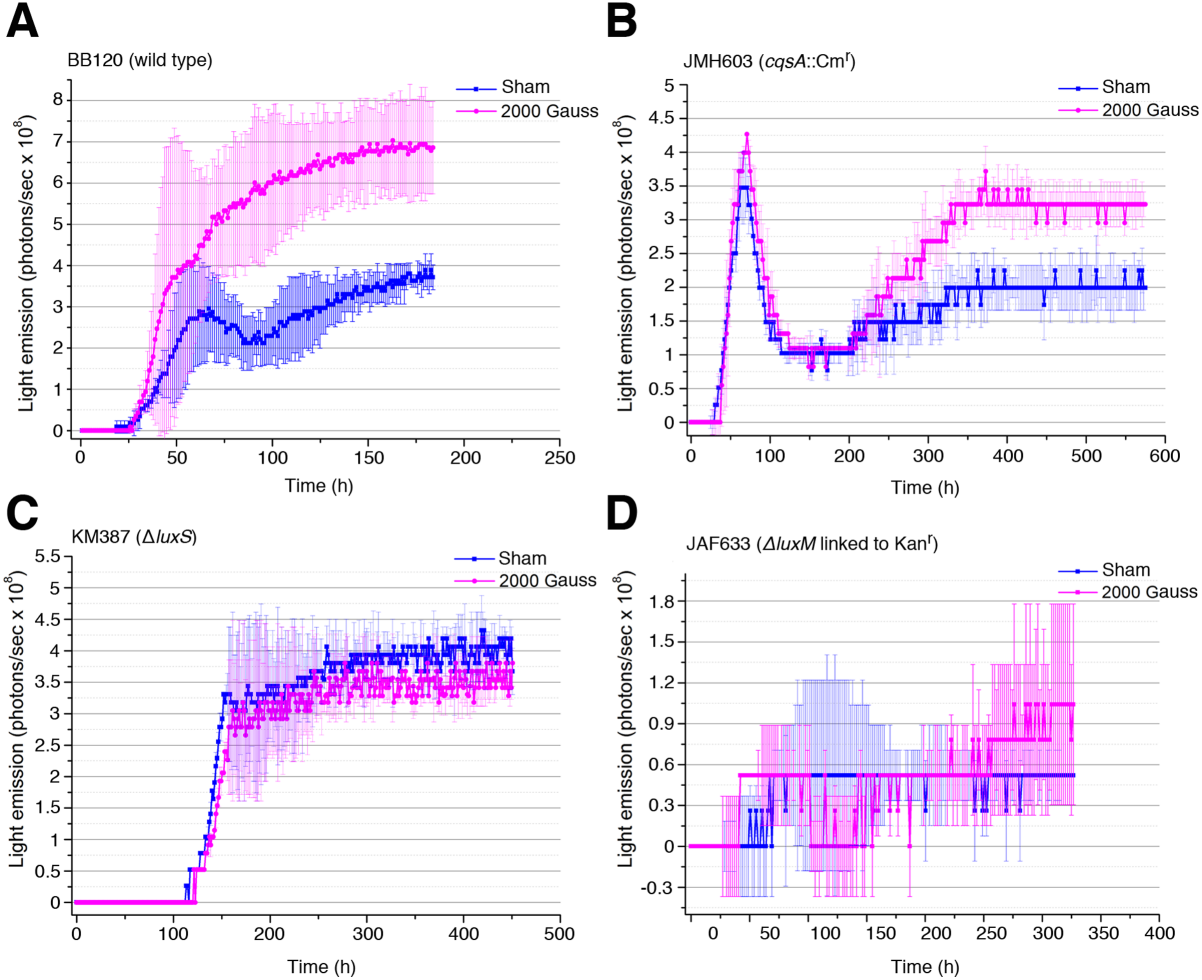
Light emission monitoring during growth of *V. harveyi* strain BB120 and derivative mutants unable to synthesize the autoinducers CAI-1 or AI-2. Harveyi clade *Vibrio* strain BB120 (A) and derivative mutants JMH603 (B), KM387 (C) and JAF633 (D) unable to synthesize, respectively, the autoinducers CAI-1, AI-2 and HAI-1 were SMF-exposed (2000 Gauss) or sham-exposed and light emission was monitored during growth on nutrient agar 1.5% containing 3% NaCl. In each panel data represent means values and standard deviations (bars) of at least three measurements.

To gain more insight about the molecular mechanisms involved in stimulation of bioluminescence by SMF, Harveyi clade *Vibrio* strain BB120 and three derivative mutants JAF633 (Δ*luxM* linked to Kan^r^), KM387 (Δ*luxS*) and JMH603 (*cqsA*::Cm^r^) unable to synthesize, respectively, the autoinducers HAI-1, AI-2 and CAI-1 were used. The temporal pattern of luminescence in BB120 was similar but not identical to that recorded in *Vibrio* sp. PS1 (more luminescent), with a rapid increase during the log phase (peaking at around 50 h) followed by a slight decrease of luminous intensity (between 50 and 100 h of growth) and a slow increase after this time (Figure 7A). When exposed to SMF (2000 Gauss), the light emission of bacteria was significantly enhanced during both the log and the stationary phases. Strain JMH603 and KM387 exhibited a different temporal pattern of luminescence (Figure 7BC), while the luminous emission of JAF633 was so low under our assay conditions that it was not possible to draw any conclusion (Figure 7D). In JMH603, the decrease of luminous intensity after the log phase was much more pronounced than in BB120, and exposure to SMF stimulated the light emission only during the stationary phase (Figure 7B). In contrast, KM387 did not exhibit the decrease of luminescence after the log phase, and was not responsive to SMF (Figure 7C) suggesting a major involvement of AI-2, the quorum sensing signal molecule containing boron.

## Discussion

### SMF affects AI-2-mediated QS

In this study, evidence is provided that PS1, a luminescent *Vibrio* strain belonging to the Harveyi clade, is responsive to SMF. When compared to sham-exposed bacteria, the light emission of SMF-exposed bacteria growing on solid medium was significantly enhanced, with a dose-response relationship, and protracted much longer during the stationary phase (Figure 3A–C and 3E). This phenomenon was neither due to an influence of SMF on bacterial growth or viability (Figure 4), nor to a direct effect of SMF on the photochemical reaction catalyzed by the bacterial luciferase. In effect, it was shown that *in vitro* bioluminescence reactions catalyzed by both bacterial [34] and by firefly luciferase [35] are not affected by SMF. Instead, our data point out an effect of SMF on QS.

Measurement of mRNA levels demonstrated that *luxR* and the *luxA* genes of the *luxCDABE* operon coding for the luciferase and the fatty acid reductase complex were significantly up-regulated in response to the SMF (Figure 5A–B). In contrast, TTS-encoding genes, whose expression was negatively affected by LuxR, were found to be down-regulated (Figure 6), suggesting that regulation of the entire QS circuit was responsive to SMF. Notably, whole genome microarray data showed that a gene homologous to *Vibrio fischeri luxR* was among the 21 genes whose expression was significantly up-regulated following exposure to strong SMF in *Shewanella oneidensis* [5], a dissimilatory iron-reducing bacterium associated with aquatic and subsurface environments and capable of inducing the extracellular precipitation of magnetite [36].

The increase of *luxR* mRNA levels in SMF-exposed bacteria was associated with decrease of amounts of Qrrs (Figure 5D), which are known to destabilize *luxR* mRNA when AIs concentrations are low [12]. This finding prompted us to speculate that the effects of SMF on QS could be mediated by production or response to some AI. Harveyi clade *Vibrio* strain BB120 produce and respond to three AIs: HAI-1 (species-specific), CAI-1 (genus-specific) and AI-2 (interspecies). The results of experiments with this strain and three derivative mutants unable to synthesize the autoinducers HAI-1, CAI-1 or AI-2 suggest that the effects of magnetic field on quorum sensing may be mediated by AI-2. Indeed, at variance with the three other strains, the AI-2-defective strain was not responsive to SMF (Figure 7). These experiments also demonstrated differences in the temporal expression pattern of bioluminescence between the strains, which may reflect different maximal activities of the three AIs during the bacterial growth, and/or a rather complex interplay between the three QS pathways [24].

The AI-2 quorum signal molecule is unique for at least two features. Firstly, unlike other AIs, which are specific to a particular genus or species of bacteria, it is produced by a large number of bacterial species serving as a “universal” signal for inter-species communication. Secondly, it has a peculiar chemical structure, a furanosyl borate diester, which remained elusive for long time [24]. Definition of the AI-2 structure and biosynthesis shined a light on the enigmatic role of boron, an element required by a number of organisms but for unknown reasons, in biology [37].

### Hypothetical biophysical mechanisms underlying the effects of SMF on AI-2-mediated QS

Based on the available literature, we could hypothesize that SMF might exert their effects on either AI-2 biosynthesis or AI-2 signaling (or both). AI-2 biosynthesis is a multistep process that starts with a transmethylation involving S-adenosylmethionine (SAM) and releasing S-adenosylhomocysteine (SAH). Then, hydrolysis of SAH by the nucleosidase Pfs yields S-ribosylhomocysteine (SRH) and adenine. SRH is then converted to homocysteine and 4,5-dihydroxy-2,3-pentanedione (DPD) by the LuxS enzyme. Finally, DPD spontaneously cyclizes to form a furanone, which is then complexed with borate to form AI-2 [38], [39]. These steps involve two enzymatic reactions that could be potential candidates for a radical-pair mechanism [2], [40].

The first candidate is the transmethylation reaction with SAM as a substrate. SAM metabolizing enzymes, indeed, are often observed in reactions involving formation of highly reactive radicals [41]. The second possibility lies in the step catalyzed by LuxS, a key enzyme in AI-2 biosynthesis. LuxS contains a divalent metal cofactor, which has been proved to be a Fe^2+^ ion that is directly involved in catalysis [39]. In particular, its catalytic mechanism [42] involves an intramolecular redox reaction, which shifts the carbonyl group from the C-1 position to the C-3 position of the ribose. Subsequent β-elimination at the C-4 and C-5 positions releases homocysteine as a free thiol. Moreover, this reaction proceeds through a reversible series of metal-bound keto/enolate intermediates and sequential proton abstraction and reprotonation involving adjacent carbon atoms. This scheme fits well with the generalizations suggested in Ref. [43] for the occurrence of radical intermediates in biological catalysis. In this context, the metal cofactor Fe^2+^ with a high magnetic moment might interact with SMF influencing the coherent spin dynamics of radical pairs intermediates [44] thereby affecting AI-2 biosynthesis. Moreover, there is some evidence that, apart from boron, DPD may complex with other ions resulting in biologically active AI-2 variants. In particular, bioluminescence was stimulated by FeSO_4_ and FeCl_3_ in boron-free medium [45]. In this context, iron could play a role also at this stage of the biosynthesis. A possible involvement of LuxS or iron containing AI-2 variants in response to SMF is consistent with the results of the experiments with the iron chelator deferoxamine that was expected to perturb the activity of iron-containing molecules (Figure 3D and 3F).

Alternatively, SMF might exert their effects on AI-2 signal transduction at the integral membrane receptor LuxPQ, comprised of periplasmic binding protein (LuxP) and histidine sensor kinase (LuxQ) subunits. In effect, due to diamagnetic anisotropic properties of biological membranes, their phospholipid molecules align and reorient in the presence of a moderate SMF thereby reducing the flexibility of the phospholipid acid chains. The consequent stiffening of phospholipid molecules increases lateral compression and thicken the bilayer thereby altering the bulk biophysical properties of the membrane, in turn affecting the activity of embedded proteins [1], [3], [46]–[47]. There is evidence that LuxP and LuxQ exist in a complex regardless of the presence or absence of AI-2. AI-2 binding causes the replacement of one set of LuxP:LuxQ contacts with another. These changes, which trigger long-range tertiary or quaternary rearrangements leading to switching in the cytoplasmic domain of LuxQ from kinase to phosphatase activity, might be affected by SMF-induced alteration in the biophysical properties of the membrane. In this context, membrane reorientation could destabilize an apoLuxP:LuxQ interface. Such a destabilization is known to increase sensitivity of the cognate receptor to AI-2 [48]–[49].

The future challenge will be to discriminate between these two mechanistic hypotheses (SMF effects on AI-2 biosynthesis or AI-2 signaling) in view of a better understanding of the biophysical bases of the stimulation of QS-regulated responses by SMF. The possible effects of AI-2 biosynthesis could be investigated *in vivo* by determining of the amount of the autoinducer produced by SMF-exposed and sham-exposed bacteria, and *in vitro* by analyzing the effects of SMF on LuxS activity using purified enzyme and substrate. The effects on AI-2 signaling could be evaluated by adding a controlled amount of exogenous AI-2 to sham-exposed or SMF-exposed *luxS*-defective *Vibrio campbellii* strain KM387.

### The magnetic inclusions of *Vibrio* sp. PS1

Another interesting issue is the presence of the electron-dense magnetic inclusions in *Vibrio* sp. PS1 (Figures 1 and 2). The results of the experiments with deferoxamine, which inhibited formation of the inclusions (Figure 1E–F) and loss of bioluminescence stimulation by SMF (Figure 3D and 3F), suggest that they may contain iron and may be involved in magnetic response. The inclusions of *Vibrio* sp. PS1 resemble those of magnetite that occur in magnetotactic bacteria. However, contrary to the magnetite crystals, they are not arranged in chains. Thus, it is unlikely that they can act like a compass needle to orient the bacteria during their movement. It should be noted that similar iron crystalline inclusions have been described in *Shewanella putrefaciens* CN32 [50]. Their size up to 50 nm is big enough to have a spontaneous magnetization and at the same time small sufficient to remain a single magnetic domain [51]. Therefore, it is conceivable that they may locally amplify the applied SMF, although the mechanism by which they may affect the behavior of bacteria when exposed to SMF is currently unknown.

### Potential biological relevance of the magnetic responses of luminous *Vibrio* strains

The most intriguing question concerns the biological significance of the observed magnetic responses of Harveyi clade *Vibrio* strains. To shed light on this aspect, we must consider what we know about the complex ecological role of these microorganisms.

As previously mentioned, luminescent *Vibrio* species are often found in symbiosis with many animals of the deep sea, which use bioluminescence as a form of optical communication for attracting mates or prey, or for defense against predation in dark environments [52]. Moreover, although several luminous strains of Harveyi clade *Vibrio* spp. can be pathogenic for aquatic organisms [53]–[54], non-pathogenic strains live in complex associations with cnidarians including corals, sea anemones, jellyfish and hydroids. These animals typically establish mutualistic endosymbiosis with photosynthetic dinoflagellates of the genus *Symbiodinium* (commonly referred to as “zooxanthellae”) [55]–[59]. In these tripartite associations bioluminescence oxygen consumption protects the animal host against oxidative stress [60]–[61]. Indeed, *Symbiodinium* photosynthesis generates high O_2_ concentration that must be kept under control to prevent the photodynamic generation of reactive oxygen species (ROS) and free radicals as a result of incomplete O_2_ reduction [62]–[64].

In this framework, it is possible that the observed magnetic responses may play some role during the host colonization, when bacteria activate the QS circuit, and bioluminescence plays a key role in the establishment and maintenance of these complex associations. The bacteria might sense a local magnetic field in the host thereby amplifying QS activation and luminescence. We do not know how a magnetic field can be generated in the host. However, it is relevant to this discussion that ferromagnetic nanoparticles (often composed of biogenic magnetite) have been detected in the tissues of a multitude of marine animals [65]–[69]. There is also evidence of magnetite and magnetic properties in algae and other photosynthetic protists including dinoflagellates [70]–[73], so that removal of algal blooms from freshwater by coagulation-magnetic separation method has been proposed [74].

If the presence of iron magnetic compounds were confirmed in the animal hosts colonized by the luminescent *Vibrio* species as well as in the symbiotic zooxanthellae living in cnidarians with the luminescent bacteria, then the local magnetic field induced by these compounds [75]–[76] might be some kind of cue the bacteria may use to sense the host environment and integrate the host signal into the QS circuit. In this regard, it is worthy of note the study of Defoirdt and Sorgeloos [77], which proposed a possible integration of a host cue into the QS circuit. This cue might be represented by the local magnetic field.

## Materials and Methods

### Bacterial strains and growth conditions

*Vibrio* sp. PS1 was described previously [15]. Harveyi clade *Vibrio* strain BB120 (wild type) and isogenic derivatives JAF633 (Δ*luxM* linked to Kan^r^), KM387 (Δ*luxS*) and JMH603 (*cqsA*::Cm^r^) [24] were kindly provided by prof. Bonnie L. Bassler (Princeton University, USA). *Vibrio* strain BB120 (also known as ATCC BAA-1116), which was originally classified as *Vibrio harveyi*, was recently proved to be *Vibrio campbellii* by microarray-based comparative genomic hybridization [78]. For bioluminescence monitoring strains were cultured on nutrient broth (Difco) containing 3% NaCl at 20°C to an optical density (O.D.) of 1.0 at 550 nm. Ten µl of the suspension was spotted at the centre of 3% NaCl nutrient agar plates and incubated at 18°C.

### Bioluminescence monitoring

To perform measurements of bacteria luminescence we prepared two identical experimental set up inserted inside the climate chamber under nearly constant temperature and humidity conditions (Figure S2). Absolute dark inside was operated. Each experimental set up contained a very sensitive photomultiplier (PMT) Hamamatsu1P28 capable to record the low intensity light emitted by our samples. Indeed the gain factor was of 5×10^6^. The nominal spectral sensibility of the photomultiplier ranged from 185 to 650 nm. Its active window, that we utilized to pick up the whole light emitted from samples, was 24 mm height and 8 mm width. Moreover, the bacterial spot was placed at a distance of 30 mm from the PMT enabling us to collect a large share of the photons emitted in the solid angle between the sample and the PMT. The photomultiplier signals were leaded to a workstation interfaced to a personal computer utilized both as storage and for timing the measurements. A channel of the workstation was also utilized to record the temperature.

### Wavelength monitoring

The wavelength monitoring was performed by a 30 cm focal length monochromator SP-308 interfaced with a PC capable to finely control the wavelength value (Figure S3). The plates containing the nutrient agar were exposed to the entrance of a UV optical fiber, which leaded the emitted light to the monochromator. The output of the monochromator was connected to a 1P28 photomultiplier. The intensity of the signal from the PMT was very low and in this case a number of photons enter the photomultiplier tube and create an output pulse train. The grating utilised had 1200 g/mm and the whole system was sensitive in the range from 350 to 650 nm. The oscilloscope shows flash signals of different intensity and repetition rate. Therefore to estimate the value of the intensity we operated the overlapping of the output pulses up to 300 samples. Recording these results on wavelength, the response was a constant value and through it we determined the wavelength spectra.

### Exposure of bacteria to static magnetic field

Exposure of bacteria to SMF was achieved by using magnets of cylindrical shape, whose south pole was applied on the back of Petri dishes as shown in Figure S4. The magnetic flux density (20, 200 and 2000 G, where 1 G = 10^−4^ T) was modulated using magnets of different magnetization. It is worth noticing that PMT should be used with care in presence of intense magnetic fields. In particular, SMFs are known to induce underestimations of the measured radiant fluxes. In order to ensure that magnets were not affecting the PMT operations, we characterized the magnetic field in the case of the 2000 G magnet and we performed several measurements to ensure a correct operation of the PMT in our setup. Such analysis, showed in Appendix 1 and Figure S6, lead us to conclude that in our operating conditions, the magnetic field reaching the PMT is not strong enough to affect measurements. Furthermore, in order to avoid any measurement artifact, the position of the SMF-exposed and sham-exposed samples in the climate chamber was switched in the different replicates.

As a further control, we verified also the effect of reversing magnet orientation. Due to the random positions of the bacteria within the spot, we expect that the effect of SMF should occur also in this case. In effect, results confirm an increased bioluminescent activity also for bacteria exposed to the north pole of the magnet (see Figure S7).

### Bacteria counting and viability assay

The Colony Forming Unit (CFU) method and the Live/Dead Bac*Light* bacterial viability kit (Molecular Probe) were used to assess viability of bacteria. Sham-exposed and SMF-exposed bacteria grown on 3% NaCl nutrient agar as described above were collected at different time intervals (50–250 h), and re-suspended in 1 ml 0.9% NaCl. The O.D. of the bacterial suspensions was measured at 550 nm. Samples were diluted so they were within the linear range of the optical system, and the O.D. data were corrected by the dilution factor used for O.D. measurement. To determine the number of CFU, and appropriate dilutions of the bacterial suspensions were placed on 3% NaCl nutrient agar plates. The plates were incubated at 20°C and the CFU number were counted after 24 h of growth. Only plates with a number of CFU ranging from 25 to 250 were considered.

For the Live/Dead Bac*Light* method bacteria were grown for 48 or 192 h. Then, they were collected and re-suspended in 1 ml 0.9% NaCl. One volume of the suspension was mixed with an equal volume of 2 X working solution of Live/Dead Bac*Light* containing a 1∶1 mixture of SYTO9 and propidium iodide. After 15 min dark incubation, 5 µl of mounted specimens were viewed with a Nikon Optiphot-2 microscope with an episcopic-fluorescence attachment (EFD-3, Nikon).

### RNA procedures

Total RNAs were extracted from bacteria sham-exposed and SMF-exposed using the RNeasy Mini kit according to the manufacturer's instructions (QIAGEN). Semi-quantitative analyses of the *luxA*, *luxR*, *hfq*, qrr, TTS-specific transcripts, normalized to 16S rRNA, were carried out by real-time RT-PCR. Total RNAs (1 µg) from MF-exposed or sham-exposed bacteria grown on 3% nutrient agar plates were reverse-transcribed by using random hexamer (2.5 µM) with Superscript RT (Invitrogen). About 0.1–1% of each RT reaction was used to run real-time PCR on a SmartCycler System (Cepheid) with SYBR Green JumpStart Taq ReadyMix (Sigma-Aldrich) and the oligonucleotide primer pairs 16Suniv-1/16S-r (specific for 16S rRNA), luxA-f/luxA-r (specific for *luxA*), luxR-f/luxR-r (specific for *luxR*), hfq-f/hfq-r (specific for *hfq*), VH02960-f/VH02960-r (specific for VH02960), VH04846-f/VH04846-r (specific for VH04846), VH05322-f/VH05322-r (specific for VH05322), VH05886-f/VH05886-r (specific for VH05886), vopB-f/vopB-r (specific for *vopB*), vopN-f/vopN-r (specific for *vopN*), vscP-f/vscP-r (specific for *vscP*). Primers were synthesized as a service by MWG-Biotech AG Oligo Production, and their sequences are reported in Table 1. RT-PCR products were 185 bp for 16Suniv-1/16S-r, 163 bp for luxA-f/luxA-r, 170 bp for luxR-f/luxR-r, 160 bp for hfq-f/hfq-r, 89 bp for VH02960-f/VH02960-r, 93 bp for VH04846-f/VH04846-r, 100 bp for VH05322-f/VH05322-r, 97 bp for VH05886-f/VH05886-r, 179 bp for vopB-f/vopB-r, 142 bp for vopN-f/vopN-r, 178 bp for vscP-f/vscP-r. Real-time RT-PCR samples were run in triplicate. The real-time PCR conditions were: 30 sec at 94°C, 30 sec at 60°C, 30 sec at 72°C for 35 cycles; detection of PCR products was performed at 83°C. The relative levels of transcripts were calculated using the 2(ΔΔC(T)) method [79].

**Table 1:** Oligonucleotides used in this study.

Name	Sequence
16Suniv-1	5′-CAGCAGCCGCGGTAATAC-3′
16S-r	5′-CTACGCATTTCACTGCTACACG-3′
luxA-f	5′-GGATAACAGCCGAGCCTTAATGG-3′
luxA-r	5′-CTCCGCGACGACATAAACAGGAG-3′
luxR-f	5′-GTGGTATCTGCCAGCGAAGAGTC-3′
luxR-r	5′-CACGCCGCGTTATTGGTGATCAAG-3′
Hfq-f	5′-CCGGTATCTATCTACCTTGTGAAC-3′
Hfq-r	5′-CACCACTGTGGTGGCTCACTGGAC-3′
VH02960-f	5′-GGACCCCTCGGGTCACCTATC-3′
VH02960-r	5′-GAAGCCAATAGGCAGTCGGATC-3′
VH04846-f	5′-CGACCCTTCTTAAGCCGAGGG-3′
VH4846-r	5′-GCAATTAGGGCGATTGGCTTATG-3′
VH05322-f	5′-GACCCTTCTTAAGCCGAGGGTC-3′
VH05322-r	5′-GCCAACCGCAATTTGTGCGATTG-3′
VH05886-f	5′-GACCCTTTTAAGCCGAGGGTC-3′
VH05886-r	5′-GCCAACCACAAGGTTTGTGATTG-3′
vopB-f	5′-GCTAACTTCGCAACACCACTCGC-3′
vopB-r	5′-GTGATGGGCGCAATCAATATGAC-3′
vopN-f	5′-CTGCTAAAGCCTTGCCTTGGC-3′
vopN-r	5′- CCAGAAAGTAAGAACTTATTGGC-3′
vscP-f	5′-CTAATCGAGCAGTCGGTAGGAAG-3′
vscP-r	5′-CCTGATCCTTTAGAGCGTTAGGC-3′

### Electron microscopy

For transmission electron microscopy (TEM) samples were fixed with 2% glutaraldehyde and 1% formaldehyde in 0.04 M piperazine-N, N'-bis (2-ethansulfonic acid) (PIPES) buffer at pH 7.0 for 2 h at room temperature. The samples were rinsed in 0.08 M PIPES buffer and twice in 0.08 M Na-cacodylate buffer and post-fixed in 1% OsO_4_ in 0.08 M Na-cacodylate buffer, pH 6.7, overnight at 4°C. Following dehydration in a step gradient of ethanol with three changes of anhydrous ethanol and one of propylene oxide incubation step at 4°C, the samples were slowly infiltrated with Epon 912 resin at 4°C, transferred to polypropylene dishes and incubated at 70°C for 24 h. Thin sections were stained with 3% uranyl acetate in 50% methanol for 15 min and in Reynold's lead citrate for 10 min and then examined with a Leo 912AB electron microscope.

For scanning electron microscopy (SEM) observations, samples were fixed with 1% glutaraldehyde, washed three times with distilled water by centrifugation, dehydrated in a graded alcohol series and critical-point dried. The sample was then mounted on Aluminum stubs, sputter-coated with gold and examined at an accelerating voltage of 20 kV with a Jeol 6060LV Scanning Electron microscope.

### Separation of magnetic crystals

Magnetic nanoparticles of *Vibrio* sp. PS1 were purified from broken cells by a magnetic separation technique. Approximately 2×10^11^ bacteria cells suspended in 5 ml of buffer A (50 mM Tris-Cl pH 7.5, 0.1 mM phenylmethylsulfonyl fluoride) were disrupted by two passes through a French pressure cell at 750 p.s.i. (1 p.s.i.  = 6.89 kPa). Unbroken cells and cell debris were removed from samples by centrifugation at 10.000 *g* for 15 min. The cell extract (1 ml) was poured into a 1 cm spectrophotometric cuvette and covered on top. Two magnets generating a magnetic field gradient were applied on opposite sides of the cuvette. Control cuvettes without magnets were used. After 12 h incubation a blackish magnetic fraction accumulated at the sides of the cuvette nearest the magnets. The nonmagnetic fluid fraction was removed by aspiration, and the magnetic phase was suspended in 500 µl of buffer A and again subjected to magnetic separation. This procedure was repeated at least three times. Finally, the magnetic particles were fixed for TEM or SEM analysis as described above.

### Statistical analysis

All data are presented as means ± standard deviations for the number of experiments indicated in each case. Statistical analysis was performed by Student's *t* test. Differences were considered statistically significant at a p value <0.01.

## Supporting Information

Figure S1 **Treatment of** ***Vibrio*** **sp. PS1 with deferoxamine.** Bacteria were plated on nutrient broth (Difco) containing 3% NaCl and incubated at 20°C in multi- well plates in the presence of different concentrations (0–500 µM) of deferoxamine as shown. Colonies were photographed after 10 to 90 h in daylight (A) or darkness (B).
(TIF)

Figure S2 **Sketch of the apparatus utilized to record the total emission light.** WS: workstations; T: thermometer; Ph: photomultiplier.
(TIF)

Figure S3 **Sketch of the apparatus utilized to record the emission spectrum.** WS2: workstation; T: thermometer.
(TIF)

Figure S4 **Detail of the experimental setup. The enclosure of the photomultiplier is made of aluminium.**
(TIF)

Figure S5 **Emission spectra of** ***Vibrio*** **sp. PS1.** Emission spectra of *Vibrio* sp. PS1after 8 (triangles), 16 (squares) and 24 (diamonds) h of growth on nutrient broth containing 3% NaCl at 20°C.
(TIF)

Figure S6 **Disk's magnetic flux density on the vertical axis, as a function of the distance from the symmetry center.**
(TIF)

Figure S7 **Light emission of** ***Vibrio*** **sp. PS1 sham-exposed or SMF-exposed over the north pole of the 2000 Gauss magnet.**
(TIF)

Table S1 **Results of real-time RT-PCR experiments.**
(DOCX)

Appendix S1 **Magnetic fields and photomultiplier sensitivity.**
(DOCX)

Appendix S2 **Effect of magnet orientation.**
(DOCX)
