# Supplementary figures and images for: Exposure to Static Magnetic Field Stimulates Quorum Sensing Circuit in Luminescent Vibrio Strains of the Harveyi Clade
Source: PLoS One. 2014 Jun 24;9(6):e100825. doi: 10.1371/journal.pone.0100825 (PMC4069165; doi:10.1371/journal.pone.0100825)

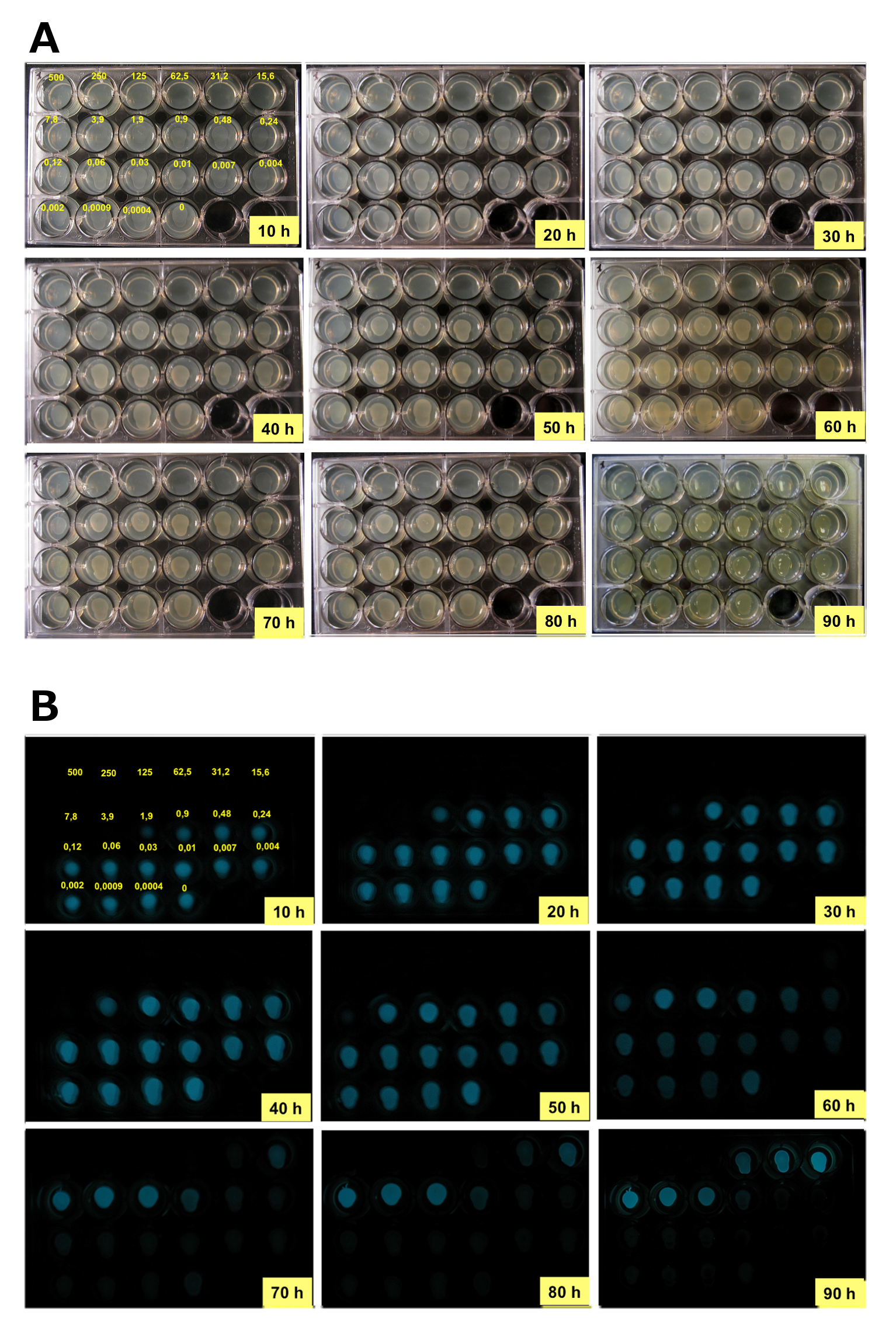

Supplement: Figure S1 — Treatment of Vibrio sp. PS1 with deferoxamine. Bacteria were plated on nutrient broth (Difco) containing 3% NaCl and incubated at 20°C in multi- well plates in the presence of different concentrations (0–500 µM) of deferoxamine as shown. Colonies were photographed after 10 to 90 h in daylight (A) or darkness (B). (TIF) [file pone.0100825.s001.tif]
