# Supplementary material for: Exposure to Static Magnetic Field Stimulates Quorum Sensing Circuit in Luminescent Vibrio Strains of the Harveyi Clade
Source: PLoS One. 2014 Jun 24;9(6):e100825. doi: 10.1371/journal.pone.0100825 (PMC4069165; doi:10.1371/journal.pone.0100825)
Supplement: Table S1 — Results of real-time RT-PCR experiments. (DOCX) [file pone.0100825.s008.docx]

**Table S1. Results of real-time RT-PCR experiments.**

| **Target RNA^a^** | **RNA Sample**  **A^b^** | **RNA Sample**  **B^b^** | **RNA Sample**  **C^b^** | **Mean (SD)** | **Mean (SD) normalized to 48 h – SE** | ***p* value^c^** |
| --- | --- | --- | --- | --- | --- | --- |
| ***luxA*** |  |  |  |  |  |  |
| 48 h - SE | 3.15 | 2.22 | 3.97 | 3.11 (0.875) | 1 (0.281) | 0.00579 |
| 48 h - MFE | 11.24 | 12.75 | 13.1 | 12.36 (0.988) | 3.97 (0.317) |  |
| 192 h - SE | 2.85 | 1.45 | 3.3 | 2.53 (0.964) | 0.81 (0.309) | 0.000457 |
| 192 h - MFE | 7.88 | 6.72 | 8.2 | 7.60 (0.778) | 2.44 (0.250) |  |
| ***luxR*** |  |  |  |  |  |  |
| 48 h - SE | 1.04 | 1.40 | 0.96 | 1.26 (0.233) | 1 (0.183) | 0.00576 |
| 48 h - MFE | 3.22 | 3.10 | 2.78 | 3.03 (0.229) | 2.39 (0.180) |  |
| 192 h - SE | 2.45 | 1.88 | 2.30 | 2.21 (0.298) | 1.74 (0.235) | 0.00870 |
| 192 h - MFE | 6.59 | 7.44 | 6.67 | 6.40 (0.467) | 5.05 (0.368) |  |
| ***hfq*** |  |  |  |  |  |  |
| 48 h - SE | 2.10 | 3.05 | 2.47 | 2.54 (0.478) | 1 (0.188) | 0.01461 |
| 48 h - MFE | 3.53 | 4.03 | 3.98 | 3.51 (0.272) | 1.38 (0.107) |  |
| 192 h - SE | 2.66 | 3.56 | 3.06 | 3.09 (0.448) | 1.216 (0.176) | 0.46161 |
| 192 h - MFE | 2.95 | 2.44 | 2.78 | 2.72 (0.262) | 1.071 (0.103) |  |
| **VH02960** |  |  |  |  |  |  |
| 48 h - SE | 14.83 | 15.67 | 16.16 | 14.55 (0.669) | 1 (0.045) | 0.00143 |
| 48 h - MFE | 4.14 | 5.12 | 4.29 | 5.71 (0.525) | 0.39 (0.036) |  |
| 192 h - SE | 17.07 | 21.43 | 19.56 | 19.35 (2.185) | 1.33 (0.150) | 0.00982 |
| 192 h - MFE | 7.98 | 9.34 | 6.78 | 8.03 (1.280) | 0.55 (0.087) |  |
| **VH04846** |  |  |  |  |  |  |
| 48 h - SE | 211.76 | 187.56 | 225.34 | 208.22 (19.137) | 1 (0.092) | 0.00396 |
| 48 h - MFE | 107.72 | 80.76 | 98.76 | 95.74 (13.731) | 0.46 (0.065) |  |
| 192 h - SE | 159.60 | 158.97 | 132.12 | 156.89 (15.686) | 0.75 (0.075) | 0.00659 |
| 192 h - MFE | 73.93 | 78.45 | 67.34 | 95.73 (5.587) | 0.46 (0.026) |  |
| **VH05322** |  |  |  |  |  |  |
| 48 h - SE | 50.85 | 44.78 | 54.89 | 43.50 (5.088) | 1 (0.116) | 0.00227 |
| 48 h - MFE | 17.44 | 14.88 | 19.65 | 17.34 (2.387) | 0.39 (0.054) |  |
| 192 h - SE | 46.17 | 42.74 | 41.89 | 43.60 (2.267) | 1 (0.052) | 0.00801 |
| 192 h - MFE | 22.36 | 25.38 | 20.87 | 22.87 (2.297) | 0.53 (0.053) |  |
| **VH05886** |  |  |  |  |  |  |
| 48 h - SE | 38.76 | 33.62 | 34.87 | 35.08 (2.683) | 1 (0.076) | 0.00938 |
| 48 h - MFE | 13.55 | 14.54 | 15.87 | 15.65 (1.162) | 0.45 (0.033) |  |
| 192 h - SE | 42.67 | 39.87 | 40.73 | 41.09 (1.434) | 1.17 (0.034) | 0.00607 |
| 192 h - MFE | 19.74 | 20.54 | 15.32 | 19.53 (2.811) | 0.56 (0.080) |  |
| ***vopB*** |  |  |  |  |  |  |
| 48 h - SE | 16.26 | 17.87 | 15.78 | 13.96 (1.095) | 1 (0.078) | 0.0000323 |
| 48 h - MFE | 5.47 | 6.89 | 4.98 | 6.77 (0.992) | 0.48 (0.071) |  |
| 192 h - SE | 24.37 | 32.02 | 28.87 | 28.42 (3.842) | 2.03 (0.275) | 0.00679 |
| 192 h - MFE | 6.83 | 8.76 | 9.23 | 8.27(1.271) | 0.59 (0.091) |  |
| ***vopN*** |  |  |  |  |  |  |
| 48 h - SE | 70.75 | 75.64 | 68.35 | 71.58 (3.714) | 1 (0.051) | 0.00103 |
| 48 h - MFE | 22.67 | 28.98 | 25.23 | 25.62 (3.172) | 0.36 (0.044) |  |
| 192 h - SE | 187.99 | 161.34 | 177.43 | 172.25 (13.420) | 2.41 (0.187) | 0.00434 |
| 192 h - MFE | 70.84 | 67.39 | 64.46 | 87.89 (3.194) | 1.23 (0.044) |  |
| ***vscP*** |  |  |  |  |  |  |
| 48 h - SE | 16.37 | 13.76 | 15.45 | 15.19 (1.323) | 1 (0.087) | 0.00685 |
| 48 h - MFE | 6.64 | 5.45 | 4.34 | 6.47 (1.152) | 0.42 (0.075) |  |
| 192 h - SE | 32.15 | 35.89 | 29.43 | 32.48 (3.243) | 2.14 (0.213) | 0.00930 |
| 192 h - MFE | 7.57 | 6.56 | 8.47 | 7.3 (0.955) | 0.49 (0.062) |  |

^a^SE, sham-exposed; MFE, magnetic field-exposed

**^b^**The RNA level of each gene in each sample was normalized to that of 16S rRNA in 1:1000 serially samples.

^c^Two-tailed Student’s t-test *p* values.
