# Supplementary material for: Exposure to Static Magnetic Field Stimulates Quorum Sensing Circuit in Luminescent Vibrio Strains of the Harveyi Clade
Source: PLoS One. 2014 Jun 24;9(6):e100825. doi: 10.1371/journal.pone.0100825 (PMC4069165; doi:10.1371/journal.pone.0100825)
Supplement: Appendix S1 — Magnetic fields and photomultiplier sensitivity. (DOCX) [file pone.0100825.s009.docx]

**Appendix S1.** **Magnetic fields and photomultiplier sensitivity.**

The producer of the photomultiplier tubes (PMT) clearly advices in its operation handbook[[1]](#footnote-1) (the “handbook” from now on) that these devices should be used with care in presence of magnetic fields. In effect, static magnetic fields are known to induce underestimations of the radiant fluxes using PMTs. This is due to the fact that a field near the dynodes could deflect the secondary electrons from their ordinary paths, leading to lower anode currents. In order to understand if the magnetic flux density coming from the magnets used during our experiments affects measurements, we should first of all have a clear understanding of the whole system (See figure S4). For the sake of simplicity, we’ll consider only the biggest field, i.e. the case of the 200 mT (2000 G) magnet.

The magnet is a permanently magnetized disk of radius *a =* 10 mm and height *L =* 5 mm. In these conditions, the magnetic flux density$B_{z}(z,r\equiv0)$outside the disk, along the vertical axis, could be expressed as

$\left|B_{z}(z,0)\right|=\frac{\mu_{0}M_{0}}{2}\left[\frac{z+\frac{L}{2}}{\sqrt{a^{2}+\left(z+\frac{L}{2}\right)^{2}}}-\frac{z-\frac{L}{2}}{\sqrt{a^{2}+\left(z-\frac{L}{2}\right)^{2}}}\right]$, (S.1)

where *μ_0_* represents the vacuum permeability while *M_0_* is the magnetization of the disk. This value has been experimentally obtained fitting with function (S.1) 7 experimental points obtained through a gaussmeter (figure S6). In particular, we obtained $M_{0}=(3.67\pm0.16)\times10^{8}\text{ A/m}$.

The Petri dishes were placed directly over the magnetic disk and the corresponding flux density at their base (i.e. at 2.5 mm from the magnet symmetry center) was $206\pm3\text{ mT}$. Moreover, considering the dish and the solid agar, the bacteria spot was at 11 mm from the base, while the PMT window was placed directly against the spot at a distance of 30 mm from it. This means that the total distance of the PMT window from the symmetry center of the magnet corresponds to 43.5 mm. At this distance the magnetic flux density at the window, estimated through the previous equation, is about 2.6 mT. If we consider that the PMT used is a side-on type, the first dynode is at about 10 mm from the window at this distance $B_{z}(z,0)\approx1.4\text{ mT}$. Close to this value, as reported in figure 13-8 of the handbook, the anode sensitivity should reduce at most of 10% for a typical side-on PMT.

Such a value is small, but could anyway affect measures. Moreover, we couldn’t determine exactly the distance of the first dynode from the window and the effect of the voltage with which we powered the PMT (0.5 kV). In effect, it is known that at higher voltage a lower effect of magnetic fields it is expected on the PMT. Consequently we performed two sets of 100 measures of the voltage signal coming from the PMT, each using the same light source (a green light emitting diode). The setup was the same used in the observations with the samples. In the first set we measured the signal coming from the PMT without positioning any magnet (free), while in the second we performed the same measurements placing the magnet (field). The signals were acquired through a digital oscilloscope (LeCroy WaveSurfer 422 ) obtaining the values (mean ± standard deviation):

$$\begin{matrix} V_{free}=44.4\pm2.5\text{ }mV \\ V_{field}=44.4\pm0.8\text{ }mV \end{matrix}$$

It is certain that the experimental observations coming from the two different conditions share the same expected value. Consequently, we could safely conclude that in our setup the presence of the magnet does not affect any measures.

The dependence of the magnetic flux density in a generic spatial point (outside of the magnet) is more tricky, but it could be resolved numerically. Direct integration for the radial and axial components of the magnetic flux density at *z =* 43.5 mm and *r =* 110 mm (the position of the PMT window on the sham-exposed sample) gives values of the same order of magnitude of terrestrial magnetism. Consequently we conclude that the disk magnet doesn’t influence the measurements also on PMT used for sham-exposed samples.

1. Available at https://www.hamamatsu.com/resources/pdf/etd/PMT_handbook_v3aE.pdf [↑](#footnote-ref-1)
