# Supplementary material for: Exposure to Static Magnetic Field Stimulates Quorum Sensing Circuit in Luminescent Vibrio Strains of the Harveyi Clade
Source: PLoS One. 2014 Jun 24;9(6):e100825. doi: 10.1371/journal.pone.0100825 (PMC4069165; doi:10.1371/journal.pone.0100825)
Supplement: Appendix S2 — Effect of magnet orientation. (DOCX) [file pone.0100825.s010.docx]

**Appendix S2.** **Effect of magnet orientation.**

It could be of interest to test if the effect on light emission is triggered also when the dishes are placed over the north pole of the magnet. Since bacteria are randomly distributed within the colony, it is reasonable to expect that the effect on bioluminescence should be noticeable also if the orientation of the magnetic field is reversed. For the sake of clarity, we performed also such experiments placing the SMF exposed samples over the north pole. Results show that stimulation of the bioluminescent emission from the exposed sample occurs also in this case (figure S7).
